# The parasitic worm-derived immunomodulator, ES-62 and its drug-like small molecule analogues exhibit therapeutic potential in a model of chronic asthma

**DOI:** 10.1038/srep19224

**Published:** 2016-01-14

**Authors:** J. C. Coltherd, D. T. Rodgers, R. E. Lawrie, L. Al-Riyami, C. J. Suckling, W. Harnett, M. M. Harnett

**Affiliations:** 1 Institute of Infection, Immunity and Inflammation, Glasgow Biomedical Research Centre, University of Glasgow, 120 University Place, G12 8TA, Glasgow, United Kingdom; 2 Strathclyde Institute of Pharmacy and Biomedical Sciences, University of Strathclyde, 161 Cathedral Street, G4 0RE, Glasgow, United Kingdom; 3 Department of Pure and Applied Chemistry, University of Strathclyde, G1 1XL, Glasgow, United Kingdom

## Abstract

Chronic asthma is associated with persistent lung inflammation and long-term remodelling of the airways that have proved refractory to conventional treatments such as steroids, despite their efficacy in controlling acute airway contraction and bronchial inflammation. As its recent dramatic increase in industrialised countries has not been mirrored in developing regions, it has been suggested that helminth infection may protect humans against developing asthma. Consistent with this, ES-62, an immunomodulator secreted by the parasitic worm *Acanthocheilonema viteae*, can prevent pathology associated with chronic asthma (cellular infiltration of the lungs, particularly neutrophils and mast cells, mucus hyper-production and airway thickening) in an experimental mouse model. Importantly, ES-62 can act even after airway remodelling has been established, arresting pathogenesis and ameliorating the inflammatory flares resulting from repeated exposure to allergen that are a debilitating feature of severe chronic asthma. Moreover, two chemical analogues of ES-62, 11a and 12b mimic its therapeutic actions in restoring levels of regulatory B cells and suppressing neutrophil and mast cell responses. These studies therefore provide a platform for developing ES-62-based drugs, with compounds 11a and 12b representing the first step in the development of a novel class of drugs to combat the hitherto intractable disorder of chronic asthma.

Asthma, a chronic ailment of the lungs associated with airway inflammation and exaggerated responses to infections, allergens and irritants, has increased dramatically in many developed countries, with prevalence highest in children^1^. However, such an increase has not been mirrored in rural areas of the Third World where parasitic helminths (worms) are highly abundant (see^2^) and this has led to the suggestion that such pathogens may protect humans from developing asthma. Although the situation is not clear-cut, being influenced by factors such as the species of helminth under study (reviewed by^3^,^4^), there is certainly sufficient supportive evidence, reinforced by the increasing body of data revealing that parasitic worms, or their secreted immunomodulatory products, can prevent the development of allergic inflammation in the lungs in mouse models (reviewed in^3^,^4^). In turn, this has generated interest in clinical trials of ‘worm therapy’ in allergy and asthma^4^ as well as in the exploitation of worm-derived immunomodulators to develop novel drugs for such diseases^5^.

Amongst the best-characterized secreted products with anti-allergy properties is ES-62, a phosphorylcholine (PC)-containing glycoprotein derived from the filarial nematode *Acanthocheilonema viteae*^5^. ES-62 directly blocks mast cell activation, thereby preventing inflammatory mediator release, both *in vitro* and also *in vivo* in the ovalbumin (OVA)-induced airway hyper-reactivity (OAH) BALB/c mouse model of acute asthma^6^,^7^. In addition, in this OAH model, ES-62 protects against cellular infiltration (eosinophils and neutrophils) and consequent inflammation-induced pathology in the lungs via blockage of Th17 responses and reversal of Th2 cell polarization. The mechanisms involved are not yet fully delineated but induction of IFNγ^+^Tbet^+^CD8^+^ T cells appears to play a key role and indeed, ES-62’s protective actions are blocked by administration of neutralising anti-IFNγ antibodies suggesting that the parasite product acts to homeostatically reset the Th cell balance in this acute airway inflammation model^6^,^8^. To date however, despite the wealth of evidence supporting the protective effects of helminth infection and worm-derived immunomodulatory products in mouse models of acute airway inflammation, clinical trials of “worm therapy” in asthma, albeit inducing some (non-significant) improvement in airway responsiveness^9^, have proved disappointing^4^. Such lack of improvement in asthma control might perhaps suggest a failure of the gastrointestinal worms tested to date to arrest or reverse pathology associated with chronic asthma such as the airway remodelling^10^,^11^ that has also proved refractory to conventional treatments despite their efficacy in controlling acute airway contraction and bronchial inflammation. Of interest given the effects of ES-62 on mast cells, there is increasing evidence that inflammatory responses triggered by the infiltration of airway smooth muscle bundles by mast cells may play a major role in airway remodelling: the resulting production of growth factors, chemokines, contractile mediators and matrix metalloproteinases are responsible for the eosinophil and neutrophil influx, increased mucus secretion and phenotype of extracellular matrix deposition that collectively contribute to the augmented airway inflammation and remodelling observed in chronic asthma^10^,^11^. Mast cells have also been reported to promote many of the parameters (cellular infiltration of the lungs and enhanced inflammation and airway hyper-responsiveness, goblet cell hyperplasia as well as increased expression of mucins 5AC and 5B and collagen deposition) associated with chronic asthma in humans^12^. We have therefore now investigated the therapeutic potential of ES-62 in a C57BL/6 mouse model in which mast cells promote chronic OVA-induced airway hypersensitivity and airway remodelling^12^.

## Materials and Methods

### Chronic asthma model

Female C57BL/6J mice (6-8 weeks old) were purchased from Harlan Olac (Bicester, UK) and group-housed at the University of Glasgow. Water and normal mouse chow were provided *ad libitum* and mice were monitored daily. All procedures were conducted in accordance with Home Office, UK animal guidelines and with the approval of the Ethical Committees of the Universities of Glasgow and Strathclyde (PPL 60/3580; PPL 60/3791; PPL 60/4300; PIL 60/10892; PIL 60/12183 and PIL60/12950). Purified, endotoxin-free ES-62 from the rodent filarial nematode *A. viteae* and its small molecule analogues (SMAs) 11a and 12b were prepared as described previously^8^,^13^,^14^. The OVA-induced model of chronic asthma was performed as previously described^12^. Briefly, female C57BL/6J mice were sensitised to OVA by intraperitoneal injection of OVA (50 μg in PBS) at days 0, 3 and 6. These mice were challenged with weekly intranasal instillations of OVA (20 μg in PBS) and, where indicated, subcutaneous injections of ES-62 in PBS (2 μg in 100 μl), SMA 11a or 12b in PBS (1 μg in 100 μl) or PBS alone at the relevant time point(s) starting from day 11 up to day 67. Unless otherwise indicated, mice were sacrificed at day 69 and serum, lungs, draining lymph nodes (DLN) and spleens were harvested, and bronchoalveolar lavage fluid (BALF) collected by cannulation of the trachea and flushing with PBS. Following fixation (10% (w/v) paraformaldehyde) and snap freezing in OCT, lung sections (7 μm) were scored blindly^6^ for disease parameters (cellular infiltration, airway thickening, mucus production and collagen deposition) on a 0–4 point scale, where 0 = normal, 1 = mild, 2 = moderate, 3 = substantial and 4 = severe, following staining with haematoxylin and eosin (H&E), Periodic Acid-Schiff (PAS), Gomori’s Trichrome or Toluidine Blue. Airway thickening was scored from all immunohistochemical staining of sections, cellular infiltration was scored from H&E and Gomori’s Trichrome sections, collagen deposition was scored from Gomori’s Trichrome sections and mucus production was scored from PAS sections^6^,^8^,^14^. The number of degranulating/degranulated and intact mast cells were calculated from Toluidine Blue sections. As multiple fields/section were scored, the mean value for each mouse was plotted and analysed. When a condition was only quantified from one staining method, between 3 and 15 fields per section were scored per mouse per time point. The phenotype and number of cells present in the BALF were determined by light microscopy^6^,^8^,^14^.

### Cytokine and antibody analysis

The levels of cytokines and other molecules in the BALF were measured by ELISA: IL-4, IL-13, MCP-1, IL-17A, IL-17F and IL-25 (eBioscience) and IL-22, eotaxin 1, eotaxin 2 and periostin (R&D systems) analysis was performed in accordance with manufacturers’ protocols. OVA-specific antibody levels in titrated serum were similarly measured by a sandwich assay, using OVA (plates coated at 20 μg/ml in PBS) and biotin-conjugated-anti-mouse IgG1, IgG2a or IgE (all from BD) antibodies, streptavidin (sv)-HRP and TMB substrate^6^,^8^.

### Flow cytometry

Flow cytometric analysis of B cell populations was performed using our previously described gating strategies^8^,^15^,^16^,^17^. Following red cell lysis, cells were counted and stained with a cocktail of antibodies for the detection of the indicated populations using anti-CD19-AF700, anti-CD23-PE/Cy7, anti-CD21-E450, anti-CD43-PE/Cy7, anti-GL7-FITC, anti-CD138-PE, anti-B220-E450, anti-B220-APC, anti-IL-10-APC, anti-CD3-FITC, anti-CD4-PerCP and anti-CD8-PE/Cy7 antibodies from eBioscience and Biolegend. Plasma cells were phenotyped as (Dump (CD3, CD4, CD8, CD11b, GR1 & CD11c)^−^ CD138^+^CD19^−^B220^−^) with the Dump^+^ markers identified using biotinylated antibodies and svPE. Alternatively, splenocytes were stimulated with 50 ng/ml PMA (Sigma-Aldrich, UK) plus 500 ng/ml ionomycin (Sigma-Aldrich, UK) and 10 μg/ml LPS (*E. coli* O111:B4, Sigma-Aldrich, UK) for 1 h before addition of 10 μg/ml Brefeldin A (Sigma-Aldrich, UK) and cells were then incubated for a further 5 h at 37 °C with 5% CO_2_. Cells were fixed and washed several times in permeabilization buffer before anti-IL-10 was added in permeabilization buffer and incubated at 4 °C for 30 minutes. Cells were then washed three times with permeabilization buffer and finally with FACs staining buffer before data were acquired using a BD LSR II flow cytometer and analysis undertaken by FlowJo software (TreeStar).

### Immunofluorescence analysis

Lung expression of MyD88 and Collagen VI was visualised by staining tissue sections (7 μm) with a polyclonal anti-human/mouse MyD88 antibody (eBioscience; 1:500 dilution) and FITC-labelled anti-rabbit IgG secondary antibody (1:200 dilution) or a rabbit polyclonal anti-Collagen VI antibody (1:200 dilution), biotinylated anti-rabbit IgG antibody (1:200 dilution) and streptavidin-AF647 (1:200 dilution), with DAPI (diluted to 1:10000 in PBS) used as a counterstain. Images were obtained using an LSM 510 META confocal laser coupled to an Axiovert 200 microscope (Zeiss) and analysed by Zeiss LSM Image Browser software.

### qRT-PCR analysis

Total RNA was extracted using an RNeasy plus kit (Qiagen) and ≤1 μg of RNA was used to synthesize cDNA (Applied Biosystems). TaqMan® RT-PCR was performed using the relevant TaqMan® Gene Expression Assays from Applied Biosystems; *periostin* (Mm00450111_m1), *muc5ac* (Mm01276718_m1), *muc5b (*Mm00466391_m1), *tlr4* (Mm00445273_m1) and *myd88* (Mm00440338_m1). Polymerase chain reactions were performed in triplicate in a StepOne sequence detector (Applied Biosystems). Data analysis was performed using the Applied Biosystems sequence detection software and the reference reporter, mouse glyceraldehyde 3-phosphate dehydrogenase (GAPDH; Mm99999915_g1), was used as an endogenous control.

### Statistical analysis

Statistical analysis was by one-way or two-way ANOVA (parametric data) or Kruskal-Wallis (non-parametric) with Bonferroni/Dunn’s post-tests or by t-test/Mann-Whitney analysis as appropriate using GraphPad Prism software. Treatments were considered significant when *p < 0.05.

## Results

### Characterisation of lung pathology in the (non-adjuvant) OVA C57BL/6 mouse model of chronic asthma

**Figure 1:**
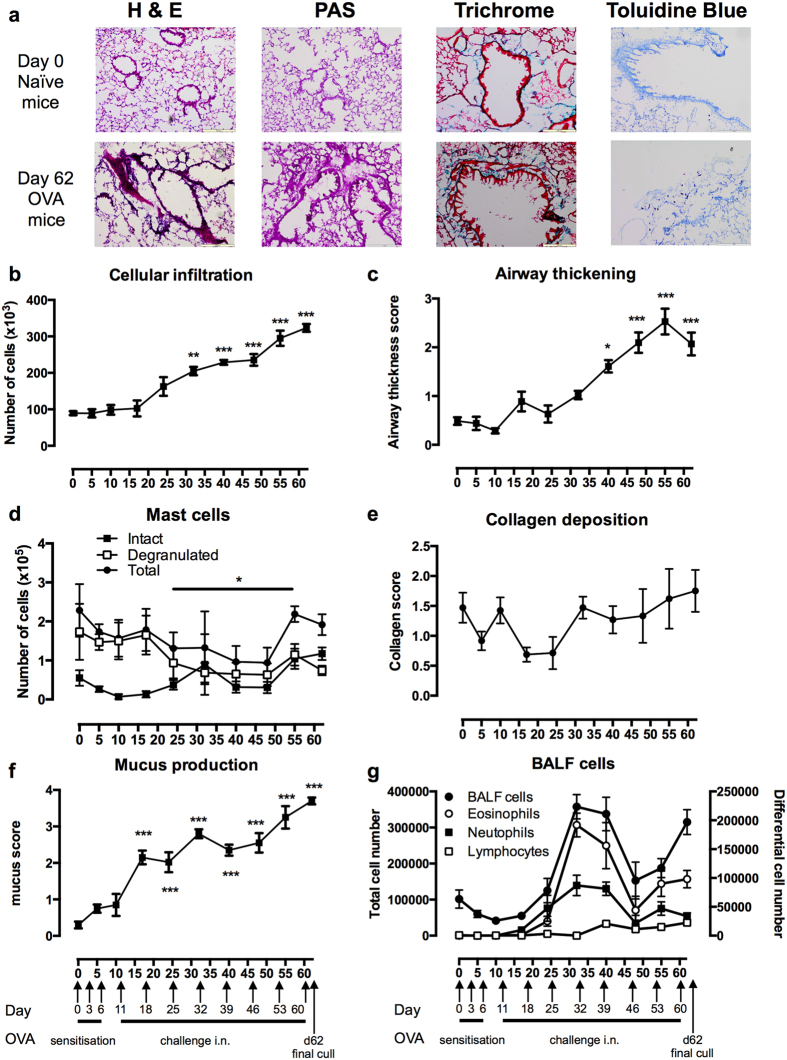
Lung pathology in the C57BL/6 mouse model of chronic asthma. (**a**) Representative images of H&E staining of airway thickness and cellular infiltration, PAS staining of goblet cells and mucus production, Gomori’s Trichome staining showing collagen deposition and Toluidine Blue staining of mast cells. Scale bars of images are 200 μm (x10 magnification). Scoring of airway disease parameters from immunohistochemical tissue sections (**b–f**) showing the means ± SEM of the mean values of individual mice where n = 5 mice culled for each time point and where *p < 0.05, **p < 0.01 and ***p < 0.001 relative to the score on day 0. (**b**) cellular infiltration; (**c**) airway thickening; (**d**) intact/degranulated mast cells; (**e**) collagen deposition; (**f**) mucus production. (**g**) Mean counts ± SEM of total BALF cells and eosinophils, neutrophils and lymphocytes in BALF where n = 5–13 individual mice culled at each time point. Total and Differential (eosinophils, neutrophils and lymphocytes) counts are scaled on the left and right hand y-axes, respectively.

The aim of the study was to establish whether ES-62 could prevent and/or arrest features of lung pathology associated with chronic asthma that have proved refractory to current treatments. Thus, the kinetics of development of associated histopathological parameters including cellular infiltration, and more specifically the presence of intact and degranulated mast cells, mucus production, collagen deposition and airway wall thickening were monitored to establish the onset of the airway remodelling characteristic of chronic asthma (Fig. 1a). Typically, cellular infiltration of the lungs, which is associated with both acute and chronic phases of asthma, was found to be elevated by d24, and to further significantly increase throughout the time course (Fig. 1b), whilst features more restricted to chronic asthma, such as airway thickening, could only be detected at later time points (d40), peaking around d55 (Fig. 1c). Mast cells could be detected throughout the time course: following a decrease in the levels of degranulated mast cells towards the end of the acute phase (d24), and perhaps consistent with their proposed role in airway remodelling, there appeared to be a late peak in their levels between d48-62 (Fig. 1d). Perhaps surprisingly, there was no significant increase in total collagen deposition detected (Fig. 1e), but this might simply reflect masking of significant changes in types such as collagen VI which have been associated with airway remodelling in chronic asthma^18^. However, reflecting induction of a chronic asthma phenotype, mucus production was found to progressively increase with time and OVA challenge (Fig. 1f).

**Figure 2:**
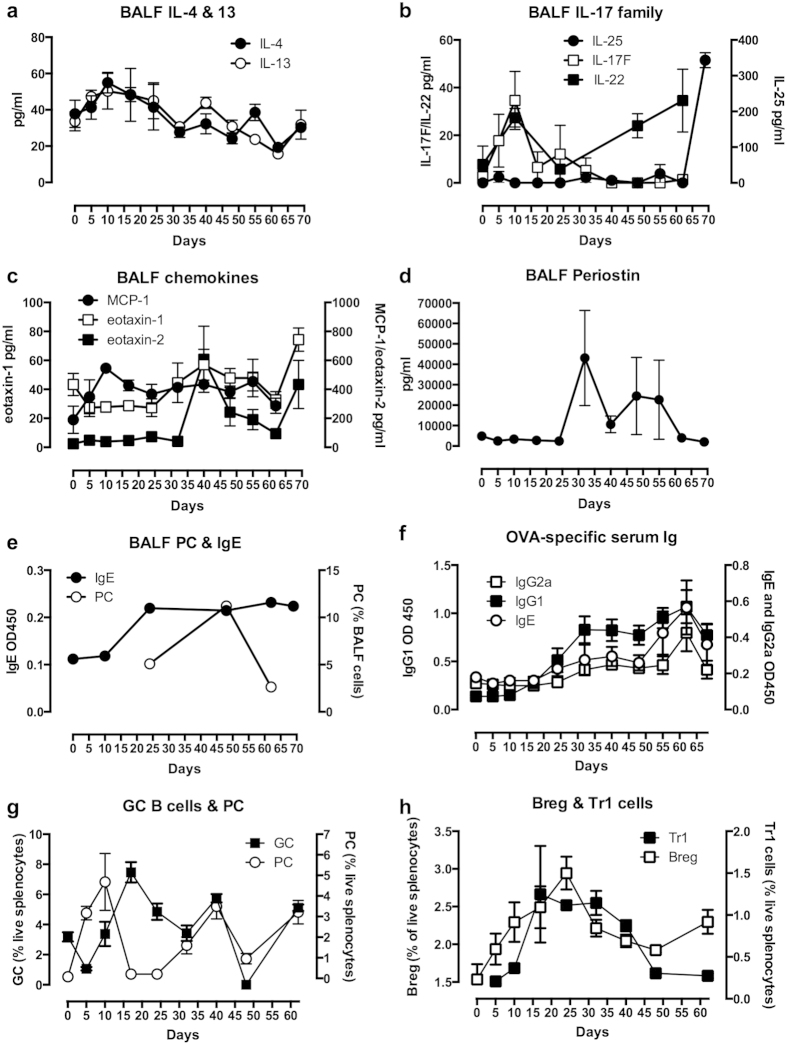
Inflammatory parameters of the C57BL/6 mouse model of chronic asthma. (**a**) IL-4 and IL-13 in the BALF are presented as mean ± SEM values of the mean levels with respect to individual mice (n = 5) at each time point; (**b**) IL-17E (IL-25; n = 5), IL-17F (n = 5) and IL-22 (n = 3) in the BALF are presented as mean ± SEM values of the mean levels in the indicated numbers of individual mice at each time point; (**c**) MCP-1 (n = 5), exotaxin-1 (n = 5–8) and eotaxin-2 (n = 5–8) in the BALF are presented as mean ± SEM values of the mean levels in the indicated numbers of individual mice at each time point; (**d**) Periostin in the BALF is presented as mean ± SEM values of the mean levels in individual mice (n = 3–5) at each time point; (**e**) OVA-specific IgE in the BALF is presented as mean ± SEM values of the mean OD450 values for individual mice (n = 3–4) at each time point whilst the levels of plasma cells (PC) in pooled samples at each time point are presented as the % of live BALF cells; (**f**) OVA-specific IgE, IgG1 and IgG2a levels in serum are presented as mean ± SEM values of the mean OD450 values for individual mice (n = 5) at each time point; levels of splenic germinal centre (GC) B cells and PC (**g**) and IL-10^+^ Breg and Tr1 cells (**h**) presented as mean % ± SEM values of individual mice (n = 5) at each time point.

Supporting the histopathology data, analysis of the bronchoalveolar lavage fluid (BALF) revealed that elevated levels of eosinophils and neutrophils could be detected by d24, peaking about d30 although there was a secondary peak around d55 coinciding with the period of airway remodelling (Fig. 1g). Preceding the primary recruitment of eosinophils and neutrophils^19^, there were early slight (reflecting the lack of Alum adjuvant) elevations in the levels of IL-4, IL-13 (IL-13 day 5, p < 0.05; Fig. 2a) and IL-17F (day 10, p < 0.05; Fig. 2b): MCP-1 production was substantially increased within days (day 10, p < 0.05), but this was generally maintained throughout (Fig. 2c). By contrast, the kinetics of eotaxin-1 and eotaxin-2 (Fig. 2c) and periostin (Fig. 2d) were more consistent with the late phase recruitment of these cell types (Fig. 1g) as well as the recently proposed role of periostin in promoting airway remodelling^20^. Levels of lymphocytes were only slightly elevated after d40 (p < 0.001; Fig. 1g), and this was perhaps reflected in the generally low levels and slow kinetics of detection of IL-17E (IL-25) and IL-22 expression in the BALF (Fig. 2b). During this period plasma cells (PC; peak d48) were also detected in the BALF (Fig. 2e) perhaps reflecting the proposed pathogenic role of IgE in asthma. Consistent with this, OVA-specific IgE could be detected in the BALF from d24 (Fig. 2e) and there was also a late serum peak observed (d49-69; Fig. 2f) typical of chronic asthma^21^. By contrast, OVA specific IgG1, but not IgG2a, was detectable by d17 with the strongly elevated levels observed between d33-69 potentially acting to perpetuate Th2-like inflammation^22^, although this pathogenic role for IgG in the form of immune complexes remains controversial in human asthma^22^. Analysis of the splenic B cell population dynamics underlying these responses revealed a primary plasma cell (PC) response during the sensitization period associated with a corresponding drop in GC cells (Fig. 2g). The PC pattern was repeated, initiating around d18 and d48, the former corresponding with the elevations in BALF IgE and serum IgG1 whilst the latter is likely associated with the late serum peaks in IgG1, IgG2a and IgE (Fig. 2f). Reflecting these effector B cell dynamics, IL-10^+^ B (Breg) and Tr1 cell populations were found to expand during the onset of pathology, presumably acting to counter-regulate the developing inflammatory response, before fading away from d24 during the established disease phase (Fig. 2h).

### ES-62 prevents development of features of airway remodelling associated with chronic asthma

**Figure 3:**
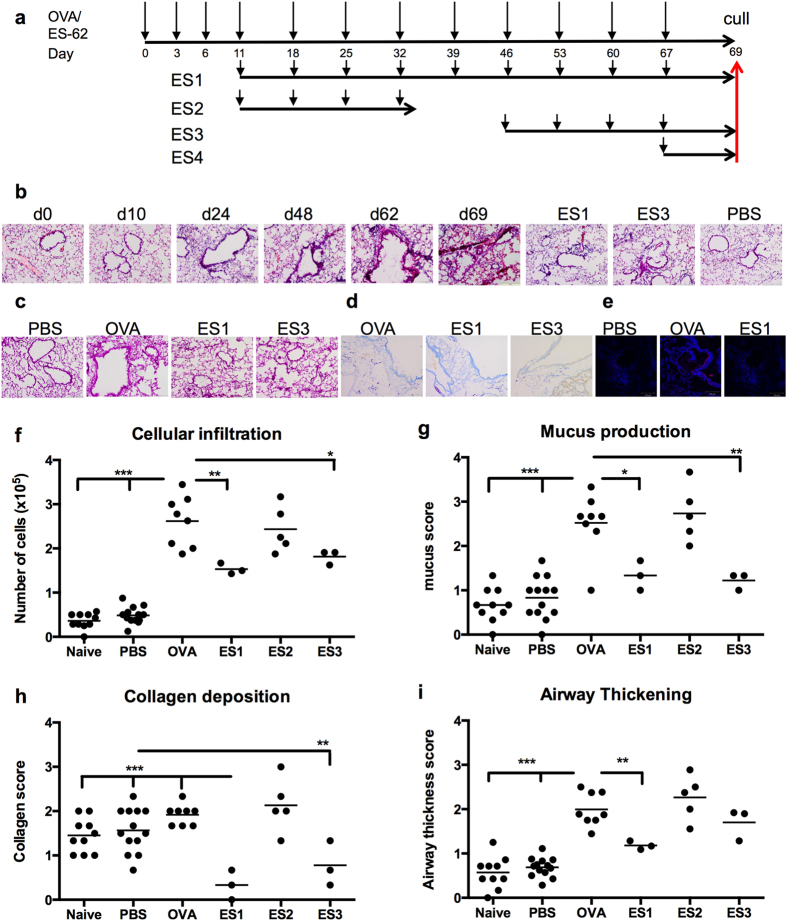
ES-62 prevents and arrests development of pathology associated with chronic asthma. (**a**) Time-course protocols of administration of ES-62 in the OVA-induced C57BL/6 mouse model of chronic asthma. (**b**) Representative images of H&E staining of lung sections at various time points during OVA-induced airway pathology and the effect of administering ES-62 weekly either from day 11 (ES1) or day 46 (ES3). Representative images of PAS (**c**) or Toluidine blue (**d**) staining of day 69 lung sections following OVA-induced airway pathology and the effects of ES1 and ES3 treatment. “PBS” represents mice that have been treated with PBS rather than OVA. (**e**) Immunofluorescence analysis of collagen VI (pink) and nuclear (DAPI; blue) staining in day 69 lung sections from the PBS-, OVA- or ES1 groups. Scoring of the effect of ES-62 treatment on airway disease parameters from immunohistochemical tissue sections (**f–i**) showing the mean values of the indicated numbers of individual mice at day 69 where *p < 0.05, **p < 0.01 and ***p < 0.001 relative to the OVA score.

Treatment of mice with ES-62 (ES1; weekly from d11; Fig. 3a) significantly reduced the cellular infiltration of the lungs (Fig. 3b,f), hyperproduction of mucus (Fig. 3c,g), mast cell infiltration (Fig. 3d) collagen, particularly Collagen VI deposition (Fig. 3e,h) and airway thickening (Fig. 3b,i) associated with chronic challenge with OVA (Fig. 1). These protective actions of ES-62 appeared unlikely to reflect generalized, sustained immunosuppression as when the parasite product was only administered weekly from d11-d32 (ES2), all protective effects were lost by d69 (Fig. 3f–i), indicating a reversible mechanism of action.

**Figure 4:**
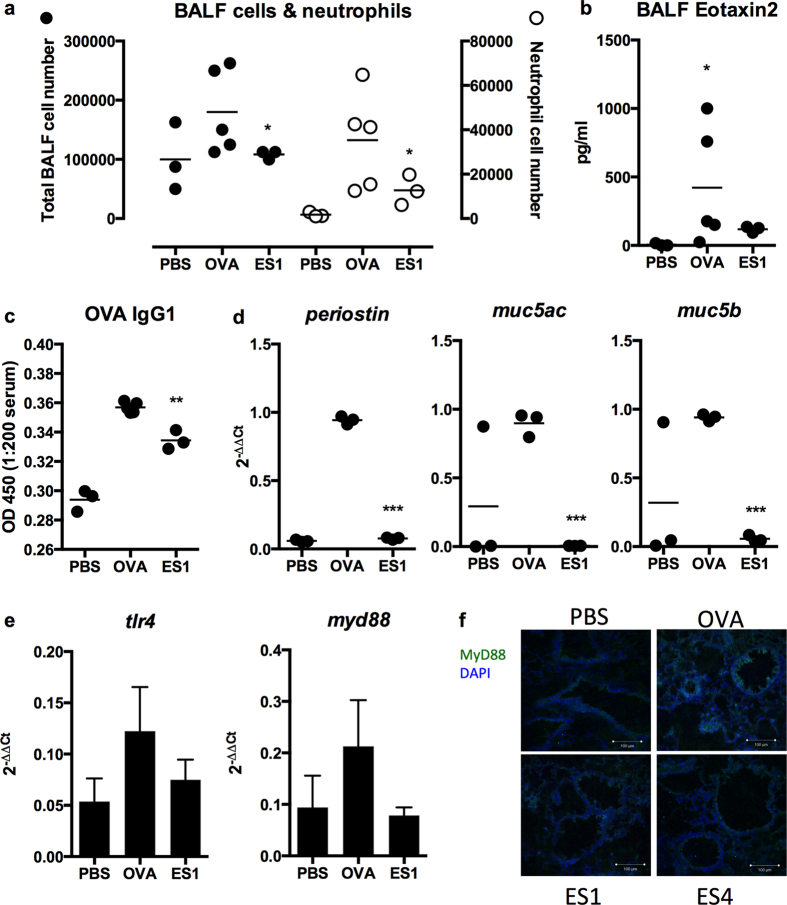
ES-62 protects against inflammation in the chronic asthma model. (**a**) Total and differential neutrophil cell counts in the BALF of the indicated number of individual mice from the PBS-, OVA- and ES1-groups at day 69, where *p < 0.05 relative to the OVA group. (**b**) Mean values of eotaxin2 in the BALF of the indicated number of individual mice from the PBS-, OVA- and ES1-groups at day 69, demonstrating that the mean ± SEM levels in the OVA, but not ES1, group are significantly (*p < 0.05) different to those in PBS-treated mice. (**c**) ES-62 reduces the levels of OVA-specific IgG1 in serum where *p < 0.05 for the mean ± SEM of mean values of the indicated number of ES1 versus OVA mice. (**d**) qRT-PCR analysis of mRNA levels of periostin, muc5ac and muc5b where data are presented as the mean of mean 2^−ΔΔCt^ values for individual mice and where ***p < 0.001 for ES1 versus OVA groups of mice. (**e**) qRT-PCR analysis of mRNA levels of *tlr4* and *myd88* where data are presented as the means ± SEM of mean 2^−ΔΔCt^ values for 3 individual mice in each group. (**f**) Immunofluorescence analysis of MyD88 (green) and nuclear (DAPI; blue) staining in day 69 lung sections from the PBS-, OVA- ES1- and ES4 groups.

The protective effects of ES-62 were further corroborated by the reduced levels of cells, specifically, neutrophils found in the BALF (Fig. 4a) and which, consistent with their expression of CCR3^23^, were associated with reductions of the CCR3 ligand, eotaxin-2 (CCL24; Fig. 4b) to levels not significantly different to those found in PBS-treated mice. Exposure to ES-62 did not suppress either BALF or serum levels of IgE (results not shown) and whilst it reduced serum IgG1 levels (at a 1/200 dilution; Fig. 4c) it did not modulate IgG2a levels (results not shown). In addition, exposure to ES-62 resulted in downregulation of expression of periostin and the mucins, Muc5AC and Muc5B in lung tissue (Fig. 4d): consistent with previously published studies showing bacterial and environmental-mediated upregulation of Muc5AC and Muc5B by respiratory epithelial cells to be TLR/MyD88-dependent^24^,^25^, we also found exposure to ES-62 acted to downregulate expression of TLR4 (Fig. 4e) and MyD88 in the lung (Fig. 4e,f), both of which have been shown to promote development of airway inflammation in response to inhaled antigen^26^,^27^ and are elevated in the OVA-, relative to PBS-treated mice.

### ES-62 can arrest established airway remodelling in chronic asthma

In addition to determining whether ES-62 could prevent development of features (airway remodelling and neutrophil infiltration) associated with chronic severe asthma, we investigated whether treatment with ES-62 could arrest or even reverse development of established airway remodelling since this phenomenon is refractory to current therapeutic regimes. As the onset of the key parameters associated with this are well established by d46 (Figs 1 and 3), we determined whether weekly treatments with ES-62 from this point onwards (ES3; Fig. 3a) prevented further subsequent development of lung pathology. This revealed that such therapeutic treatment arrested the further development of cellular infiltration (Fig. 3b,f), mucus hyperproduction (Fig. 3c,g), collagen deposition (Fig. 3h) and airway thickening (Fig. 3i) and indeed, appeared to reverse some of the histopathology evident at the start of the treatment (Fig. 3b): although scoring of the sections did not show a significant reduction in airway thickening in relation to the OVA group, some degree of improvement was evident as these ES3 scores were not significantly different from those resulting from the “prophylactic” ES1 treatment (Fig. 3i). Collectively, these data suggest that therapies based on ES-62 may be able to protect against future deterioration of lung function or even reverse an established chronic asthma lung phenotype somewhat.

### ES-62 ameliorates allergen-induced neutrophil exacerbations in chronic asthma

**Figure 5:**
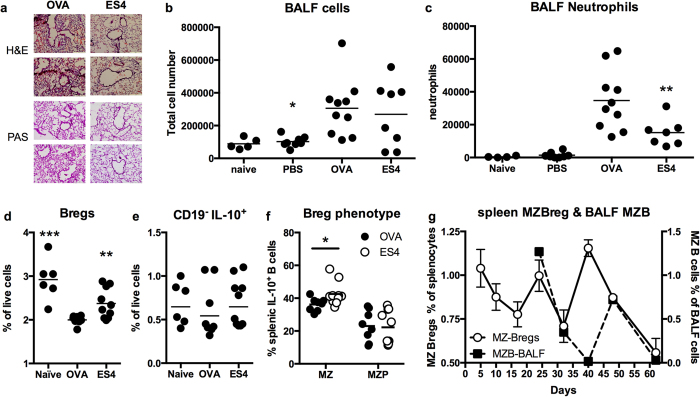
ES-62 ameliorates OVA-induced inflammatory exacerbations. (**a**) Representative images of H&E and PAS staining of day 69 lung sections from OVA- and ES4 groups of mice. Total (**b**) and differential neutrophil (**c**) cell counts in the BALF of individual mice in the indicated treatment groups where in (**b**), the mean ± SEM value of the OVA, but not the ES4, group is significantly (*p < 0.05) different to that of the PBS mice whilst in (**c**), the levels of neutrophils in the ES4 group are significantly reduced (**p < 0.01) relative to those found in OVA mice. The levels of splenic IL-10^+^ Breg (**d**) and CD19^−^IL-10^+^ lymphocytes (**e**) presented as the % live cells in individual mice (n = 5) and where for the mean ± SEM values, **p < 0.01 and ***p < 0.001 relative to that of the OVA group. In (**f**), the levels of MZ (CD21^+^CD23^±^) and MZP (CD21^+^CD23^+^)-like Bregs are presented as the % of IL-10^+^ B cells in the spleen of the indicated number of individual mice and where for the mean ± SEM values, *p < 0.05 relative to that of the OVA group. (**g**) Levels of splenic MZ Bregs presented as mean % ± SEM values of individual mice (n = 5) at each time point. Levels of MZ-like B cells in pooled samples from each group at the indicated time points (from d24) are presented as the % of live BALF cells.

Inflammatory flares consequent to repeated exposure to allergen are a debilitating feature of severe chronic asthma. Thus, to investigate whether in addition to arresting airway remodelling ES-62 can act therapeutically to ameliorate such exacerbations, we tested the effects of administering it immediately prior to the final challenge with OVA (ES4; Fig. 3a). This revealed that whilst, as expected, this treatment had no substantial immediate effect on mucus production or airway thickening (Fig. 5a), it reduced cellular infiltration (mean score ± SEM: PBS, 2.618 ±0.2; ES-62, 1.995 ±0.19: p ≤ 0.05). Reflecting this, although the total cellularity of the BALF in the ES4 group was not significantly different from that of the OVA control mice (Fig. 5b) there was a significant decrease in the levels of neutrophils (Fig. 5c), but not macrophages, lymphocytes or eosinophils (results not shown), recruited. However, this inhibitory effect on neutrophil infiltration was not associated with significant suppression of the levels of the Th2 (IL-4 and IL-13)- or Th17 (IL-17A, -E, F or IL-22)-associated cytokines, chemokines (eotaxin-1, -2 or MCP-1) or the inflammatory mediator, periostin (results not shown) implicated in the recruitment of pro-inflammatory cells to the airways during asthma pathogenesis. Rather, this ES-62-mediated amelioration of OVA-induced neutrophilic exacerbation was accompanied by a downregulation in MyD88 expression in the lung (Fig. 4f) and restoration of the putative regulatory IL-10-producing splenic B cell population (Bregs) back towards the levels observed in naive mice (Fig. 5d). By contrast, this ES-62 treatment had no effect on the levels of splenic CD19^−^IL-10^+^ lymphocytes (surrogate for regulatory T cells, particularly Tr1 cells; Fig. 5e), a finding perhaps consistent with the ES-62-mediated restoration of CD19^+^IL-10^+^ levels reflecting an increase in the MZ (CD21^+^CD23^±^), but not MZP (CD21^+^CD23^+^), -like Bregs (Fig. 5f) that have been reported to be involved in the IL-10-, but not regulatory T cell-dependent protection afforded in models of acute OVA-induced AHR following infection with the parasitic trematode worm, *Schistosoma mansoni*^28^. ES-62 had no effect on any of the other proposed phenotypes of Breg (Follicular, B1, B10 and plasmablast) analysed (results not shown).

These data therefore suggested that ES-62 might act to reset the homeostatic balance of effector: regulatory B cells that normally acts to resolve aberrant airway inflammation. Consistent with this, determination of the levels of MZ-like Bregs in the spleen (Fig. 5g), showed that in contrast to the total levels of Bregs (Fig. 2h), which progressively rise during the initiation phase of disease (peaking at d25) presumably as a homeostatic response to increasing inflammation, MZ-like Bregs decrease during this period (trough at d17), displaying an essentially inverse pattern to that of GC B cells (Fig. 2g). Likewise, their sustained reduction from d40-onwards precedes the late rise in splenic GC B cells and plasma cells observed at d62 in the spleen. Moreover, analysis of BALF levels of MZ-like B cells (as a phenotypic surrogate of MZ-like Bregs in the airways) from d24 showed their profile to generally shadow that of MZ Bregs in the spleen (Fig. 5g): collectively, these data perhaps suggest a particular correlation between decreases in MZ Bregs and airway inflammation that ES-62 targets to prevent development of pathology associated with chronic asthma.

**Figure 6:**
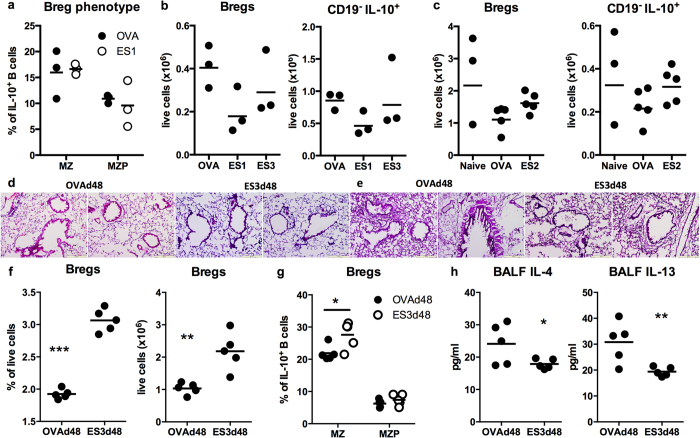
Induction of IL-10 Bregs is an acute response to ES-62 treatment. (**a**) Levels of MZ (CD21^+^CD23^±^) and MZP (CD21^+^CD23^+^)-like Bregs are presented as the % of IL-10^+^ B cells in the spleen in individual ES1-treated mice (n = 3). (**b**) The levels of splenic IL-10^+^ Breg and CD19^−^IL-10^+^ lymphocytes in individual ES1, ES3 or OVA mice (n = 3). (**c**) The levels of splenic IL-10^+^ Breg and CD19^−^IL-10^+^ lymphocytes in individual naive, OVA or ES2 mice (n = 5). Representative images of H&E (**d**) and PAS (**e**) staining of lung sections from the OVAd48 and ES3d48 groups. (**f**) The levels of Bregs presented as the % live cells in individual mice (n = 5) and where for the mean ± SEM values, **p < 0.01 and ***p < 0.001 relative to that of the OVA group. In (**g**), the levels of MZ (CD21^+^CD23^±^) and MZP (CD21^+^CD23^+^)-like Bregs are presented as the % of IL-10^+^ B cells in the spleen in individual mice and where for the mean ± SEM values, *p < 0.05 relative to that of the OVA group. (**h**) Mean values of IL-4 and IL-13 in the BALF of the indicated number of individual mice from the OVAd48 and ES3d48 groups, demonstrating that the mean ± SEM levels in the ES3d48 group are significantly (*p < 0.05 and **p < 0.01) different to those in the OVAd48 mice.

We next investigated whether the protection afforded by the prophylactic ES1 and therapeutic ES3 (post-onset of lung pathology associated with chronic asthma) interventions also reflected resetting of IL-10^+^Bregs levels: analysis at d69 cull suggested that prophylactic treatment with ES-62 (ES1) did not modulate the levels of either MZ- or MZP-like Bregs (Fig. 6a) and indeed, if anything each of the ES1 and ES3, but not the ES2, treatments appeared to decrease the levels of CD19^+^IL-10^+^ (Bregs) and CD19^−^IL-10^+^ lymphocytes (Fig. 6b,c) in the spleen at this time. However, analysis of a variant (ES3d48) of the ES3 treatment in which lung pathology and Breg responses were examined (relative to their OVA controls) at d48 rather than at d69 revealed that whilst expected, this intervention had essentially no effect on lung pathology in this short time period (Fig. 6d,e), as with ES4 treatment the ES3d48 group showed elevated proportions and numbers of IL-10^+^Bregs (Fig. 6f), specifically MZ-like Bregs (Fig. 6g) and this immunomodulation was associated with reduced levels of IL-4 and IL-13 (Fig. 6h) but not eotaxins, MCP-1 or periostin in the BALF (results not shown: no IL-17 or IL-22 could be detected in these airway samples).

### Small Molecule Analogues (SMAs) of ES-62 target neutrophil exacerbations associated with allergen challenge

**Figure 7:**
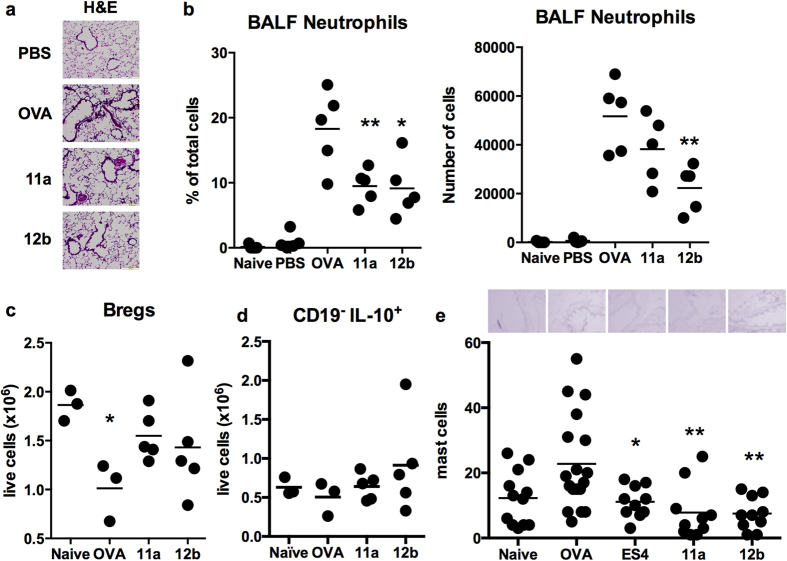
SMAs 11a and 12b ameliorate OVA-induced inflammatory exacerbations. (**a**) Representative images of H&E staining of lung sections from the indicated groups of mice. (**b**) The proportions and numbers of neutrophils in the BALF of individual mice in the indicated treatment groups where *p < 0.05 and **p < 0.01 relative to the mean ± SEM value of the OVA group. The levels of splenic IL-10^+^ Breg (**c**) and CD19^−^IL-10^+^ lymphocytes (**d**) presented as the numbers of live cells in individual mice (n = 5) and where for the mean ± SEM values, in (c) the OVA group only is significantly different (*p < 0.05) from the control PBS group. (**e**) Representative images of toluidine blue staining of lung sections from the indicated groups of mice and the quantitative analysis of the numbers of mast cells in the lungs of individual mice from the indicated treatment groups and where data represent the mean numbers of mast cells/section for each mouse, with *p < 0.05 and **p < 0.01 relative to that of the OVA group.

As an immunogenic molecule, ES-62 is itself probably unsuitable as a therapeutic: however, we have recently generated a library of small molecule analogues (SMAs) based on its active PC-moiety^13^, two of which, 11a and 12b can mimic the immunomodulatory activity of ES-62 and suppress allergic inflammation in models of acute airway^6^,^8^,^14^ and skin hyper-reactivity^6^,^29^. SMAs 11a and 12b were therefore tested for their ability to suppress the inflammatory exacerbation resulting from the final OVA challenge in this chronic asthma model: as with ES-62, such therapeutic treatment with 11a and 12b resulted in reduced cellular infiltration of the lungs (Fig. 7a) that reflected a significant reduction in the levels of neutrophils in the BALF (Fig. 7b) and was accompanied by restoration of the levels of IL-10^+^ B cells in the spleen to that seen in naive mice (Fig. 7c). Moreover, as observed with ES-62, SMAs 11a and 12b had no effect on the levels of splenic CD19^-^IL-10^+^ lymphocytes (Fig. 7d). Rather, such therapeutic treatment of mice with SMAs 11a or 12b immediately prior to the final OVA challenge, like ES-62 resulted in reduction of the levels of mast cells detected in the lungs (Fig. 7e).

## Discussion

Asthma is often described simply as a Th2-polarised eosinophilic inflammatory disease with effector mast cell hypersensitivity: however, increasingly it is recognised as a complex, heterogeneous disease encompassing multiple phenotypes including Th2-independent, neutrophilic subgroups that are typically associated with severe and/or smoking-related asthma as well as those refractory to corticosteroid treatment^30^. Moreover, unlike acute airway contraction, the mucus hyper-production and airway obstruction and remodelling associated with chronic asthma appears to be independent of bronchial inflammation. Rather mast cell infiltration of, and interactions with, airway smooth muscle (ASM)^31^ promotes mast cell survival and activation and their differentiation towards a fibroblastoid phenotype^10^,^11^,^32^, as well as airway hyper-reactivity (AHR) and ASM remodelling^10^,^11^,^32^. The inflammatory microenvironment arising out of ASM-mast cell myositis drives mucous gland hyperplasia, fibrosis, collagen deposition and airway thickening, resulting in reduced airway function^32^. Whilst acute bronchial contraction caused by eosinophilic inflammation can be controlled by steroids, the irreversible airway remodelling associated with chronic severe asthma is refractory to such conventional therapies^33^. Thus, there has been increasing interest in developing new and more personalised therapies to counter the various subtypes of this increasingly economically important group of diseases^30^,^34^.

Whilst the incidence of asthma has been rising dramatically in the industrialised world mirroring the control of infectious disease in these regions, epidemiological evidence from developing countries suggests that infection with helminths may offer some protection against allergic disease: this component of the Hygiene Hypothesis^35^ is further supported by health initiatives like deworming being reflected by recent increases in prevalence of atopy in the tropics^36^. This evolving “story” has therefore recently focused interest *both* on the potential for therapeutic exploitation of such parasitic worms, or their immunomodulatory products, in allergy and asthma *and* questioned the wisdom of mass deworming programmes to eradicate helminth-associated morbidity^36^,^37^,^38^. However, despite the wealth of supporting studies in experimental models of airway inflammation, clinical trials investigating the efficacy of ‘worm therapy’ in allergy and asthma have proved disappointing to date^4^,^9^,^36^. This perhaps reflects that such therapeutic worm-induced immunomodulation may only target the Th2-mediated inflammation causing bronchial contractions in acute disease and exacerbations, rather than improving lung function by arresting/reversing pathology associated with chronic asthma. Interestingly, therefore, we found that in addition to suppressing Th17/Th2-mediated initiation of allergic airway inflammation, ES-62 could also suppress neutrophilic airway inflammation and desensitise mast cell responses in acute BALB/c OVA/alum-induced airway inflammation^6^,^7^,^8^. Importantly, exposure to ES-62 could maintain survival of all such treated mice whilst 4 out of 6 PBS-treated mice died by d57 in a chronic version^39^ of this eosinophilic/Th2 model, modified such that the BALB/c mice continued to receive OVA intranasally weekly from d21-56 (Kean & Harnett, unpublished). To determine whether the mechanisms underpinning such protection involve modulation of airway remodelling, we have now investigated whether ES-62 exhibits therapeutic potential in a C57BL/6 model of chronic asthma^12^, in which mice are sensitised to OVA without the use of ALUM adjuvant to avoid hyper-Th2 skewing of the immune response, and then challenged weekly with OVA intranasally (until d67) to mimic the pattern of repeated allergen exposure occurring during human disease^12^. Certainly, such mice develop several of the features associated with chronic severe human asthma including mast cell-dependent lung infiltration by lymphocytes, monocytes, eosinophils and neutrophils, mucus production and airway remodelling^12^.

Repeated prophylactic treatment with ES-62, starting prior to intranasal OVA challenge, indeed suppressed development of airway remodelling and was associated with reduced cellular, specifically neutrophilic, infiltration of the lungs, OVA-specific IgG1 production, mucus (muc5ac and muc5b) hyperproduction and, consistent with the observed suppression of periostin production^23^, airway thickening. The precise mechanisms involved remain to be established but as such functional modulation was accompanied by partial reduction of TLR4 and MyD88 expression in the lungs, this protection presumably reflects, at least in part, the dampening of TLR/MyD88-driven airway inflammatory and bronchoconstriction responses^26^,^27^,^33^,^40^,^41^, particularly as MyD88 signalling has been implicated in promoting neutrophil recruitment^33^,^41^ and development of plasma cells^16^,^42^,^43^. In addition, most importantly, weekly administration of ES-62, even after airway remodelling had been established, was found to at least prevent any further deterioration suggesting that the parasite product can reduce the airway pathology associated with chronic asthma. Moreover, the inflammatory exacerbations resulting from repeated allergen exposure that are a serious complication of chronic asthma were ameliorated by treatment with ES-62 acting to reduce both neutrophilic and mast cell infiltration of the airways, when administered immediately prior to OVA challenge during the chronic phase. Intriguingly, this protection is associated with an increase in the levels of B cells with the capacity to produce IL-10 and hence putatively, act as regulatory B cells (Bregs): collectively, this is reminiscent of the effects observed with therapeutic allergen-induced tolerance (AIT) where mast cell responses are suppressed within hours and Bregs are induced within days to effect protection^44^,^45^,^46^. Bregs can act directly and/or via the induction or functional restoration of IL-10-producing Tr1 and FoxP3^+^ regulatory T cells to suppress mast cell, neutrophil and eosinophil responses and survival as well as reset effector B and Th cell responses^44^,^45^,^46^,^47^ and in AIT, some induced Bregs can develop into IgG4-producing cells that act to block IgE-mediated mast cell responses^44^,^45^,^46^. Certainly, regulatory B and T cell populations have been reported to be reduced or dysfunctional in atopic subjects^44^,^45^,^46^,^48^,^49^ and consistent with this, although both Bregs and Tr1 cells appear to increase to counter the acute initiating inflammatory response, they decline prior to the onset of pathology associated with airway remodelling in our mouse model of chronic asthma. Intriguingly, it has recently been recognised that certain “helper” neutrophil populations can prime B cell responses and antibody production^50^ and hence Breg-mediated downregulation of neutrophil responses could also potentially contribute to the observed ES-62-mediated suppression of pathogenic OVA-specific IgG1 production.

Indeed, protection against allergic and autoimmune disease by helminths is increasingly recognised as involving the mobilisation of Bregs and also, in some cases, consequent regulatory T cell responses^38^,^49^,^51^,^52^,^53^,^54^. In terms of asthma, in acute eosinophilic (alum adjuvant) models of OVA-induced AHR, the TLR7-elicited T2-MZP-like Bregs associated with *S. mansoni* infection were found to be induced in the spleen, but not the lung or lung-draining mediastinal lymph nodes (MLN) of mice and transfer of splenic CD1d^hi^ B cells conferred protection against AHR in recipient mice in an IL-10- and regulatory T cell-dependent manner 49,51,52. Similarly, with respect to the MZ-like Breg population induced by *S. mansoni*, only those cells isolated from spleens conferred protection in an IL-10-dependent manner although in this case, regulatory T cells did not play an essential role but rather only promoted suppression of pathology: by contrast, such Bregs harvested from the lungs acted in an IL-10- and Treg-independent manner whilst those from MLN did not confer any protection^28^,^49^,^55^. Consistent with these data, we found that the protection afforded to mice undergoing OVA-induced AHR with ES-62 or the SMAs 11a and 12b during the intranasal challenge phase of the acute alum model^8^,^14^ is associated with increased levels of IL-10^+^Bregs in the spleen (unpublished results). We now show here that ES-62 also acts to dynamically induce Bregs, particularly those of the MZ phenotype to counter inflammation throughout the development of pathology associated with chronic asthma. Moreover, we have previously shown that the protection afforded by ES-62 against the development of autoimmune arthritis and lupus-like nephritis in mouse models^16^,^17^ is likewise associated with homeostatic resetting of the effector:regulatory B cell balance and that this switch reflects a partial downregulation of MyD88 expression^16^: however, as seen here also, restoration of the levels of Bregs to those pertaining in naive healthy mice was not accompanied by a corresponding increase in regulatory Tr1 cells, but at least in the case of AHR this may reflect the targeting of MZ Bregs which do not appear to require regulatory T cells for their action in suppressing lung pathology^28^. Given that ES-62 therefore generally appears to target inflammation by homeostatically resetting resolution of inflammatory responses, it was at first sight rather surprising that rather than detecting an increase in Bregs in the ES1 or ES3 treatment groups at d69, if anything but presumably reflecting their suppression of pathology, the ES1 and ES3, but not ES2, treatments resulted in a reduction in the levels of Bregs. However, as with the ES4 treatment, the increase in MZ-like Bregs in the ES3d48 group suggests induction of Bregs to be an acute response to ES-62 (and its SMAs) in AHR, that acts to reset the homeostatic balance of inflammation and its resolution that is defective in chronic asthma, either in terms of the levels or functionality of Bregs^48^,^49^,^56^.

In conclusion, as ES-62 can reduce the airway pathology associated with chronic asthma and ameliorate inflammatory exacerbations arising from repeated exposure to allergen, these studies provide a platform for developing ES-62-based drugs or those that mimic its actions. To this end we have generated a library of small molecule analogues based around the active PC-moiety of ES-62, and we have previously shown two of these, 11a and 12b, to mimic its ability to desensitise mast cell responses and afford protection against acute neutrophilic airway inflammation and allergic skin inflammation^14^,^29^. Now, as SMAs 11a and 12b have both been shown to mimic ES-62 in targeting mast cell, neutrophil and Breg responses during inflammatory exacerbations and hence potentially, the myositis contributing to airway remodelling in this mouse model, they represent the first step in the development of a novel class of drugs to combat the hitherto intractable pathology associated with chronic asthma as well as that of steroid refractory patients.

## Additional Information

**How to cite this article**: Coltherd, J. C. *et al*. The parasitic worm-derived immunomodulator, ES-62 and its drug-like small molecule analogues exhibit therapeutic potential in a model of chronic asthma. *Sci. Rep.* **6**, 19224; doi: 10.1038/srep19224 (2016).
